# Taking Bacteriophage Therapy Seriously: A Moral Argument

**DOI:** 10.1155/2014/621316

**Published:** 2014-04-29

**Authors:** Gilbert Verbeken, Isabelle Huys, Jean-Paul Pirnay, Serge Jennes, Nina Chanishvili, Jacques Scheres, Andrzej Górski, Daniel De Vos, Carl Ceulemans

**Affiliations:** ^1^Laboratory for Molecular and Cellular Technology, Burn Wound Centre, Queen Astrid Military Hospital, Bruynstraat 1, 1120 Brussel, Belgium; ^2^Faculty of Pharmaceutical and Pharmacological Sciences, KU Leuven, Herestraat 49, 3000 Leuven, Belgium; ^3^Department of Behavioural Sciences, Royal Military Academy, Renaissancelaan 30, 1000 Brussel, Belgium; ^4^Centre for Intellectual Property Rights, KU Leuven, Minderbroedersstraat 5, 3000 Leuven, Belgium; ^5^Burn Wound Centre, Queen Astrid Military Hospital, Bruynstraat 1, 1120 Brussel, Belgium; ^6^R&D Department, Eliava Institute of Bacteriophage, Microbiology and Virology, 3 Gotua Street, 0160 Tbilisi, Georgia; ^7^University Hospital Maastricht, Maastricht University Medical Centre, P.O. Box 5800, 6202 AZ Maastricht, The Netherlands; ^8^Dept. Hospital Organization, National Institute of Public Health NIZP, ul. Chocimska 24, 00-791 Warsaw, Poland; ^9^Dept. Medical Microbiology, University Medical Centre Groningen, Hanzeplein 1, 9713 GZ Groningen, The Netherlands; ^10^Institute of Immunology and Experimental Therapy, Polish Academy of Sciences, ul. Rudolfa Weigla 12, 53-114 Wrocław, Poland; ^11^The Medical University of Warsaw, Żwirki i Wigury 61, 02-091 Warsaw, Poland

## Abstract

The excessive and improper use of antibiotics has led to an increasing incidence of bacterial resistance. In Europe the yearly number of infections caused by multidrug resistant bacteria is more than 400.000, each year resulting in 25.000 attributable deaths. Few new antibiotics are in the pipeline of the pharmaceutical industry. Early in the 20th century, bacteriophages were described as entities that can control bacterial populations. Although bacteriophage therapy was developed and practiced in Europe and the former Soviet republics, the use of bacteriophages in clinical setting was neglected in Western Europe since the introduction of traditional antibiotics. Given the worldwide antibiotic crisis there is now a growing interest in making bacteriophage therapy available for use in modern western medicine. Despite the growing interest, access to bacteriophage therapy remains highly problematic. In this paper, we argue that the current state of affairs is morally unacceptable and that all stakeholders (pharmaceutical industry, competent authorities, lawmakers, regulators, and politicians) have the moral duty and the shared responsibility towards making bacteriophage therapy urgently available for all patients in need.

## 1. Introduction

### 1.1. Factual (Nonnormative) Observations concerning Bacteriophage Therapy

The excessive and improper use of antibiotics has led to an increasing incidence of bacterial resistance and a significant threat to human health [1, 2]. Yearly, more than 400.000 people are infected by multidrug resistant bacterial strains, often called “superbugs” [3]. Superbugs have a considerable economic impact: extra hospital costs and related productivity losses amount to more than 1.5 billion Euros per year within the European Union (EU). In the United States (US), infections with multidrug resistant bacteria cause 20 billion US$ in additional health care costs and 35 billion US$ societal costs annually [4].

At the same time, it is becoming more and more difficult and expensive to develop new antibiotics as an adequate response to the phenomenon of multidrug resistance. Actually, very few new antibiotics are in the pipeline of the pharmaceutical industry at the moment [5].

Early in the 20th century, bacteriophages were described as entities that can control bacterial populations. Bacteriophages (or “phages”) are viruses that infect and replicate within specific bacteria without harming others. Although bacteriophage therapy (hereafter BPT) was developed and practiced in Europe and the former Soviet republics, the western world abandoned the use. This was mainly because at that time (1930s) there was a lack of knowledge of what a bacteriophage really was (a virus) as well as the discovery of antibiotics. These molecules are well-characterized chemical substances, relatively easy to produce in a well-controlled fashion, initiating the golden age of antibiotics, the so called “miracle drugs” [6].

Given the worldwide antibiotic crisis, the existing and continued experiences build up in Eastern Europe and the former Soviet Republics combined with recent encouraging animal and human study results; there is a growing interest for BPT in modern medicine and the agrobioindustry, a recognized potential reservoir for antibiotic resistant germs [1, 3, 7–10].

Despite this growing interest, introducing BPT in the western medical world remains highly problematic as a consequence of* four main obstacles*. First, historical clinical data about the safety and effectiveness of BPT are not considered proven and validated by European regulators. Second, given the substantial costs and investment in the development and marketing of conventional medicinal products by the pharmaceutical industry, there is in our actual pharmacoeconomic model an imperative demand for a strong intellectual property (IP) protection. For now, such protection is rather fragile for natural lytic phages. Third, an efficient and effective BPT-concept needs to be flexible and tailored to the patient [11, 12]. That requirement is not compatible with the usual timeframes (years) for the development and the marketing of conventional medicinal products [11, 12]. Rather, the regulatory framework for medicinal product development, as present in most countries, calls for drugs to have a fixed chemical composition. Bacteriophages challenge this definition by being mutable. Last (fourth obstacle), uncertainty exists about the potential negative coevolutionary consequences of unlimited use of BPT [13]. In view of these obstacles, access to BPT for patients in need remains highly problematic as discussed earlier [12, 14].

Recently, Henein emphasized that so far no bioethical bacteriophage therapy debate has been published. He gave the most recent* E. coli* 0104 outbreak as an example [15]. Indeed in 2011, an emerging strain of O104:H4* Escherichia coli* caused a serious outbreak of food borne haemolytic uremic syndrome and bloody diarrhoea in Germany. Antibiotics were of questionable use and 54 deaths occurred, beside tens of clinical cases with lasting sequels [16, 17]. Several bacteriophage research groups had in their collection isolated candidate therapeutic bacteriophages that efficiently lyse the* E. coli* O104:H4 outbreak strain [18–20]. The public health sector never asked for these phages during the outbreak and none of the scientific papers published during the outbreak mentioned BPT as a potential treatment. Nestlé Research Centre even offered their phage isolate to the German public health sector during the epidemic, but the proposal was apparently not addressed [20].

Międzybrodzki et al. [21] addressed briefly the ethical aspects of bacterial drug resistance and phage therapy. The authors also highlighted the appeals for decisive changes in the policies governing the development of antimicrobials. Bacteriophages should be considered as a public good and the government should be responsible for their development and production. Thus, the development and introduction of new antimicrobials should not only be regulated by market forces [21].

The main purpose of this paper is not so much to fleece out these four main obstacles, nor to determine how an adequate regulatory framework for BPT might look like. This has already been done in other publications [11, 12, 14, 22]. We here argue why there exists a moral need or duty to develop such a regulatory framework. The different actors in the field, mainly the industrial partners, politicians and regulators as well as consumers, urgently need to take up their responsibility in order to guarantee BPT accessibility for patients in need. What is more, when the costs of phage therapy and antibiotic therapy were compared, phages were approximately 50% cheaper than antibiotics. This means that a wider application of phage therapy could lead to substantial savings in healthcare costs and make antibacterial therapy accessible to those who otherwise cannot afford treatment [23].

### 1.2. Normative-Ethical Considerations concerning BPT

Given the above nonnormative specifications of BPT, the basic moral problem associated with BPT can be formulated as follows. BPT, when used in a flexible (tailor-made, locally developed) and sustainable manner, has the potential of saving thousands of lives every year [11, 24]. However, due to the above-mentioned obstacles, access to that therapy remains highly problematic in the western world. How can we argue from a moral point of view that this situation is simply unacceptable?

Central to this moral problem are the preservation and restoration of the health and well-being of the patient. Two basic underlying moral principles are relevant in this patient-centred approach and will be further investigated in this paper. The first is the* principle of nonmaleficence,* which implies the obligation not to inflict harm on another. This principle is designed to protect the patient. The second principle is the* principle of beneficence,* which implies the obligation to prevent or to remove harm or the obligation to promote good [25].

## 2. Bacteriophage Therapy: An Ethically Justified Medical Therapy?

The fact that efficacy of BPT has not yet been proven according to European regulatory standards is one of the obstacles that clearly suggests the moral relevance to investigate the* principle of nonmaleficence* in the context of BPT. Indeed, a patient has a right not to be subjected to a medical treatment or therapy that has not yet been rigorously tested for its effectiveness and possible health risks (as is BPT). The moral duty, however, not to subject a patient to a not yet approved therapy is not an absolute, but a* prima facie duty*. This means that under well-defined, specific circumstances this duty not to subject someone to a not yet approved therapy can be set aside [26]. At this point in the argument, it is appropriate to introduce the notion of an “*Ethically Justified Medical Therapy*” (EJMT). This is a medical therapy that has not yet obtained an official approval for its health effectiveness (at least not according to western standards), and/or for which some doubts remain concerning possible health risks, but the use of which seems to be morally acceptable given the specific circumstances of the case at hand. For such a therapy to be labelled as an EJMT, six criteria need to be met (Table 1) [27].

First of all, there has to be a* just cause* or a very good reason for subjecting a patient to a possibly hazardous medical therapy and/or a therapy the effectiveness of which has not yet been demonstrated in a rigorous manner. The moral weight of whatever it is we want to achieve with this therapy has to be sufficiently important. In the case of BPT-therapy we might think of a patient whose life or limb is threatened by a serious bacteriological infection. Saving that patient's limb or threatened life, for instance, constitutes such a good reason beyond any doubt.

Secondly, we need to make sure that all the moral agents involved are motivated by* ethically proper intentions*. In a clinician-patient relationship, the medical practitioner ought to have the intention to help the patient in need, although other interests (e.g., hospital-related) may play a certain role as well. Therefore, if indeed a patient's health is at stake (a good reason), then our intention for using the yet unapproved therapy has to be about improving the patient's health condition and not about commercial, research, or cost-reducing benefits.

Next, there needs to be a* reasonable chance* that the use of the therapy in a particular case will have the desired result. In case of BPT, based on preliminary examinations and testing by experienced specialists, multiple case studies reporting success were published [28–30].

Efficacy of bacteriophage therapy has not yet been proven according to European regulators, but anyway the concept of reasonable chance does not imply real proof of such efficacy. What is more, there must be a good prospect that the probable health benefits will outweigh the risks of subjecting the patient to the therapy (*proportionality*). It is important in this respect to try to avoid as much as possible undesired side effects related to BPT [31–33].

Another criterion is that the unapproved therapy needs to be a* last resort*. All existing treatments must have been tried with little or no success. BPT might for instance be considered as such a last resort when marketed antibiotics are no longer effective. In reality, in particular circumstances of multidrug resistance, BPT offers today a* reasonable and feasible* alternative or complimentary treatment approach to save such patients. Indeed it has been shown that BPT and traditional antibiotic therapy can create a synergic beneficial effect [34, 35].

Medical practitioners' assessment in these circumstances is crucial. It needs to be stressed that in clear cases of antibiotic resistance, BPT most likely constitutes the first and not just the last resort. Indeed, the last resort principle only demands that we consider reasonable alternatives, since hopeless situations due to lack of adequate alternatives need to be avoided as much as possible according to international and national legislations and declarations. In Belgium, the compassionate use regime provides such a “last resort” mechanism, whereby a medical practitioner may apply an unapproved product provided marketing authorization is applied for or clinical trials are ongoing. At the international level, Article 37 of the Declaration of Helsinki states the following:* “In the treatment of an individual patient, where proven interventions do not exist or other known interventions have been ineffective, the physician, after seeking expert advice, with informed consent from the patient or a legally authorized representative, may use an unproven intervention if in the physician's judgement it offers hope of saving life, re-establishing health or alleviating suffering. This intervention should subsequently be made the object of research, designed to evaluate its safety and efficacy. In all cases, new information must be recorded and, where appropriate, made publicly available”* [36]. Note however, that the Declaration of Helsinki, although at the international medical community level is well accepted as a basic document, is not a national binding law, meaning that it has no national juridical value and as such could put the practitioner in a position of juridical vulnerability as experienced in France (personal communications, M.D. A. Dublanchet and Court Lawyer B. Papin).

Finally, a decision to subject a patient to a not yet approved therapy needs to be made in respect with the patient's right to autonomy (*legitimate authority*) [26]. However, it should be noted that a patient's consent to be subjected to a not yet approved treatment is not a sufficient condition to go ahead with the therapy. The other above-mentioned criteria need to be respected as well. Within this context, we need to determine whether a patient's (or its legal representative) consent constitutes a necessary condition.

Although these six criteria were originally developed within another context, namely, that of the just war theory (JWT), it seems that these criteria can also be of valuable use in the ethical discussion on BPT [37]. This is because the underlying ethical argument is very similar. The JWT starts from the supposition that war is a moral evil, and that because of this we have an obligation to avoid war as much as possible. Notice here again that the obligation not to wage war is not an absolute, but a* prima facie duty*. In specific circumstances (determined by the above criteria) war can be a morally acceptable option (for instance in case of self-defense or humanitarian intervention). A very similar moral mechanism is at work in our discussion about BPT. Subjecting a patient to a not yet approved therapy is morally wrong, but sometimes, in specific (exceptional) circumstances (determined by the six criteria), it can be a morally* acceptable* option to subject a patient to such unapproved therapy, and not just a* morally excusable option*.

Whether or not BPT is an EJMT needs to be checked on a* case-by-case* basis. Numerous types of bacterial infections exist. Any manner to combat such infection needs to be considered separately. In fact, medical ethical committees provide also case-by-case reasoning while evaluating proposals for treatment.

We would like to conclude the first part of our argument by saying that the six criteria we used to evaluate BPT's moral permissibility form the ethical basis for some kind of* precautionary* regulatory framework. Until it can be adequately shown that BPT can live up to western health care standards, there will be a constant need to justify the use of BPT on a case-by-case basis. Moreover, given the specific nature of BPT (its interactive and ever-evolving character) and the need to focus on tailor-made solutions, it will be difficult, if not impossible, to claim once and for all (as it is probably the case for traditional “static” chemical medicinal products) that BPT is effective and safe. This would also imply that any regulatory framework designed to assure the safety and effectiveness of flexible and sustainable BPT will never completely lose its precautionary character.

## 3. Towards a Moral Duty to Invest in the Development of Bacteriophage Therapy

### 3.1. Do Pharmaceutical Companies Have a Duty to Care?

Demonstrating that BPT constitutes an EJMT in a sufficient number of cases is of course but a first part of our moral argument. Showing that in a specific case BPT is an EJMT makes its use* morally permissible *in that specific case. But showing* moral permissibility* for BPT is only a* necessary condition* for our purpose, which is demonstrating that it is simply morally unacceptable to obstruct (or, at least, to not sufficiently facilitate) the accessibility to BPT. We also need to show that somehow there exists a* moral duty* to take the necessary steps in order to make flexible and sustainable BPT available in a more organized fashion.

Pharmaceutical companies are the primary actors in the business of developing new and improved medicinal products and therapies. Hospitals do not have the financial capacity and resources to fully develop bacteriophage-based products according to the current pharmaceutical medicinal product guidelines. Although our initial focus will be on pharmaceutical companies, this does not mean that other actors (like public authorities) are absolutely absolved of all responsibility in this respect. We will come back to this specific issue later on in this paper.

In our patient-centred approach, we perceived that the principle of* nonmaleficence* provides a so-called* prima facie* moral protection for the patient against therapies and treatments that have not yet proven their health effectiveness and/or for which some doubts remain concerning their possible health risks. In trying to establish a moral duty to contribute to the development of lifesaving therapies, such as BPT [28, 30, 38], it is suitable to turn to the other basic moral principle in bioethics, that of* beneficence*.

Despite the fact that it is not in the interest of the classic pharmaceutical industry, organized in accordance with the actual pharmacoeconomic environment, to invest in sustainable BPT (see the above mentioned obstacles), the beneficence-principle seems to provide a sufficient moral basis for arguing that the pharmaceutical industry has a duty to do so anyway.

In the 1970s, most supporters of market economy embraced Friedman's view that the social responsibility of business is to increase its profits, not to relax the conditions of profit-maximization on behalf of the wider interests of society [39]. But, is this acceptable when it comes to healthcare? Surely, companies involved in the healthcare industry should live up to their responsibilities towards the public interest, not only towards their shareholders. To quote Blasszauer: “*medicine is a moral enterprise whether it is practiced in the system of slavery or market economy*” [40]. Defenders of Friedman's thesis claim that for executives to use company resources to advance social goals, it would be for them to usurp the political function. In this context it might thus be up to the political world to demand healthcare companies to defy the laws of economics and fulfil social duties [12, 41].

One of the reasons why the above conclusion is not that straightforward has to do with the specific nature of the beneficence-principle. Indeed this basic principle is especially morally relevant within the relation between the health care professional and his patient. Because of his specific role, it can be said that the health care professional has the moral obligation to undertake all the necessary and reasonable measures to improve his patient's health condition (or to prevent his patient's health from deteriorating). The relational context between a patient and the pharmaceutical industry is of course very different from the one between the health care professional and his patient. It is no longer a relation of care, compassion, and beneficence, but a relation of an economic or a commercial kind. The industry's role is to develop, produce, and sell medicinal products, whereas the role of the patient is that of a consumer. This does not mean that the pharmaceutical industry has absolutely no obligations whatsoever towards its patient-consumers. The industry has the obligation to take all the necessary and reasonable measures to ensure the effectiveness and the safety of the medicinal products it decides to develop, produce, and sell [42]. Within that same commercial relation (“patient-consumer/pharmaceutical industry”) it is very hard, as far as we can tell, to justify the imposition of an additional requirement on the pharmaceutical industry to invest in the development and production of medicinal products that are of a lifesaving importance to some patients, but that are rather uninteresting from a pure commercial point of view as is the case for flexible and sustainable BPT that has been shown lifesaving in specific individual cases [28, 30, 38]. Here we need to look for another way to morally justify an obligation to make available for as many patients as possible medicinal products or therapies for which there does not seem to exist a sufficient commercial incentive to start a development and production process.

### 3.2. Do Pharmaceutical Companies Have a Social Responsibility to Invest in BPT in Order to Promote Overall Social Welfare?

Perhaps a more promising line of argument is of a* utilitarian kind*. One might quite convincingly argue that economic actors in our society, like private businesses and companies, do not only have specific client-related obligations, but also a somewhat broader social responsibility to promote overall well-being in society. If we agree that this is the case (that there exists such a utilitarian-based responsibility), then it still remains to be seen of course whether the moral requirement to promote novel antimicrobial approaches like BPT can indeed be based on such a notion of social responsibility. To verify this, we need to explore two separate questions.

The* first question* goes as follows: to what extent may we assume that the promotion of BPT (eventually in combination with existing therapies) constitutes indeed the course of action that, compared to other possible alternatives, will lead to better results in confronting the health challenges related to an increasing antibiotic resistance problem in bacteria (hereafter the resistance problem)? Proving that BPT is the best option in tackling this specific health problem from a utilitarian point of view is essentially a* technical matter* that requires further thorough knowledge in microbiology and in health care economics. In other publications it has been shown that this may be the case [7, 43, 44].

Establishing with a reasonable degree of certainty that BPT is indeed a good option in confronting the resistance problem from a utilitarian point of view constitutes, however, a necessary, but not a sufficient condition for our purpose to demonstrate that there is such a thing as a moral requirement to promote BPT. A part of our common-sense morality clearly states that promoting the best overall result does not automatically generate a moral requirement to do so. We can illustrate this with an example. Suppose that ten people are on the verge of losing their lives (that they are about to drown). Suppose also that person A could save all of them if he wanted to. The only trouble is that person A can only do so with a considerable risk to himself. Although, saving the ten lives (even with the risk of losing his own life in the process) is the best option from a utilitarian point of view; there is a general agreement within common-sense morality that person A is under no moral obligation to do so. In this specific example person A is allowed to favour his own life, even if sacrificing it in order to save the ten others would be preferable from a utilitarian viewpoint. In the theory of normative ethics, common-sense morality contains so-called agent-favouring options (or agent-favouring prerogatives) [45]. These are moral principles that protect an agent from the obligation to* always* promote the overall good. The key word here is “always”.

Indeed, in some cases the promotion of the overall good does create a moral requirement to do so. For instance: if person A could save the lives of those ten people with practically no risk for himself, he can no longer avoid the obligation to do so. In order to understand the mechanism of the agent-favouring options better, it is essential to take into account two factors: the (probable) cost to the agent, and the (probable) amount of overall good or well-being that is at stake. If the amount of good at stake outweighs the cost to the agent (who has no risk to lose his life), then the requirement to promote the overall good can no longer be blocked by the agent-favouring option. This seems to suggest that the option protecting a moral agent's self-interest is characterized by some kind of threshold, the level of which is determined by the cost the moral agent will have to pay when he decides to serve the overall good. Once the amount of good or well-being at stake crosses that threshold, the creation of a moral requirement to bring about this amount of good can no longer be blocked by the agent-favouring option. A general rule seems to be then: the higher the cost to the agent, the higher the threshold, the stronger the agent-favouring option, and the lesser the probability that the agent will be subjected to the obligation to promote the overall good. It should be clear, however, that whenever the interests of an agent are protected by such an agent-favouring option, he or she is still at liberty to promote the overall good. If, for instance, person A is a heroic kind of a person and he will not hesitate to save those ten people, even if this means sacrificing his own life. It is obvious that such an act will not be morally condemned. Far from it: such an act will typically be praised and admired. But again, it is not an* obligatory* act, but rather a* supererogatory* act inspired by idealism [46].

Let us assume for the sake of argument that such agent-favouring options are indeed a part of our common-sense morality. This being the case, it might very well be that the economic and commercial interests of the pharmaceutical industry are going to be protected by such options. Even if it would appear that promoting BPT is indeed the best choice in tackling the resistance problem, pharmaceutical enterprises cannot be morally obligated to sacrifice their own commercial interests in order to give priority to the development of BPT. It is possible, however, that some pharmaceutical companies could decide to go ahead anyway with the development of BPT. But, again, such a decision would constitute a supererogatory act, not a mandatory act.

We now come to our* second question*: is it reasonable to assume that there exists such an agent-favouring option that protects the private interests of the pharmaceutical industry and thereby blocks the creation of a moral requirement to promote BPT? Answering this question requires a more profound cost/benefit analysis. In case of pharmaceutical companies' investments in bacteriophage product development, costs are considerable since all regulatory processes need to be conducted [20]. Assuming that (a) cost-based option(s) protecting the interests of a moral agent is (are) indeed a part or our common-sense morality and (b) the development of BPT will definitely entail a commercial costs to a pharmaceutical company (resources that will be invested in this kind of research and development can no longer be used to develop more lucrative products); it is also fair to assume that pharmaceutical companies will try to protect their private interests by appealing to such agent-favouring option and pretend that there is no such thing as a duty to promote BPT. Whether or not the pharmaceutical industry is justified in hiding behind such agent-favouring option will depend on two factors: what is the* moral force* of that option and will it be strong enough to block a moral requirement to promote BPT? At this point in the argument we will need to evaluate the importance of the cost to the pharmaceutical industry. This will indeed give us some idea of the moral force of the agent-favouring option (the higher the cost, the stronger the option). Today, any organization, pharmaceutical company, or any nonprofit actor like a hospital, willing to implement BPT, is expected to follow the classical marketing authorization and market placement procedures. The costs for developing BPT following these frameworks can be as much as 400–800 million USD [15] and not realistic for nonprofit actors.

In addition, intellectual property rights (IPRs) that in general provide owners exclusivities to recoup investment costs are hardly unavailable (or weak) in the context of BPT [15]. The reason is that IPRs do not protect natural products (e.g., natural bacteriophages) or processes covering natural phenomena (e.g., mechanism of action of BPT based on the inherent coevolution of bacteria and bacteriophages). However, given the widespread availability of bacteriophages and the fact that natural bacteriophages are from a structural point of view less complex than other biological (protein-based) products or advanced therapies medicinal products, the costs to develop BPT could eventually be lower compared with these latter medicinal products.

Will the overall health benefits of saving thousands of lives, generated by the introduction of BPT, cross the option's threshold (the height of which is determined by the option's moral force)? To verify this, we need to compare the costs of investment in BPT development by pharmaceutical companies with the unproven potential BPT of, for example, savings of the lives of 25.000 Europeans each year [28, 30, 38], with the cost associated with bacterial outbreaks caused by bacterial infections [47], costs to the social security for hospitalized patients [3], and emotional costs associated with the disease itself. According to the authors of this paper, these costs to society by not developing BPT clearly indicate that the option's threshold protecting the pharmaceutical industry's private interests will be crossed. In our opinion, pharmaceutical companies hence do have a moral duty to contribute in one or another way to the development of BPT. Thinking of phage therapy as a sustainable antibacterial approach should have the potential of cost reduction for society that should be considered as a major incentive for companies to invest in BPT. However, in the current regulatory climate, the pharmaceutical companies are unable to do it without adequate incentives or support. Clearly other stakeholders like the public authorities need to provide the right incentives by creating feasible regulatory frameworks.

## 4. Closing the Moral Gap

### 4.1. Reconciling Private Interests and Social Responsibilities

According to the authors, previous analysis demonstrates that arguments can be found to support a moral requirement for the pharmaceutical industry to promote BPT. It remains to be seen what specific form this moral requirement will take. The pharmaceutical industry, due to its specific knowledge and know-how, will certainly have some role to play in the validation process of the clinical data relating to BPT (obstacle (1)) and/or in furthering the research concerning the potential negative coevolutionary consequences of unlimited use of BFT (obstacle (4)). In addition, it is important to consider how in the future industrial companies will maintain (keep on respecting) this moral duty and how these companies can be made aware of the importance of creating and maintaining high moral standards in the long run. History portrays similar developments, more specific in the domain of cell and gene therapy. Several regulatory incentives, specific types of subsidies and models for public-private partnerships have contributed to stimulate the industry for investments into these therapies [48]. More in particular, a specific European framework covering advanced therapy medicinal product (Regulation 1394/2007) was created, offering legal incentives (e.g., scientific advice at reduced costs) to developers of cell and gene based therapies. Specific calls for research funding were launched via the FP7 programs of the European Commission, and large-scale collaborative efforts are in place between industry and academia, under the umbrella of, for example, Europe's Innovative Medicines Initiative (IMI).

### 4.2. Other Stakeholders' Responsibilities

Until now we have solely focused on the pharmaceutical industry as the principal bearer of this potential moral requirement to promote BPT. Obviously, political authorities, much more so than private companies, have a social responsibility to promote public health in the most efficient way they can. What is more, due to their public nature, public authorities cannot hide behind cost-based options to protect their “private” interests as private companies can. As such, since we have shown that according to the evidence available, the promotion of flexible (characterized by its locally produced and tailor-made nature) and sustainable BPT is, compared to other alternatives, for different particular disease situations, the best solution to promote overall health benefits, then there is a public moral duty to do so. This particular public requirement to promote this specific kind of BPT can perhaps best manifest itself by providing an adapted regulatory framework, so that the full potential of BPT as a locally prepared and tailor-made therapy can finally be realized.

## 5. Conclusion

In this paper, we have argued that the current state of affairs as described in the introduction is morally unacceptable. We succeeded in underpinning the desirability for developing a flexible and sustainable BPT-adapted regulatory framework with the necessary moral force. The authors are aware that moral arguments in favour of BPT may equally be identified (via a similar moral analysis) and apply to other areas of drug development (e.g., orphan diseases). We argued that the pharmaceutical industry has a moral duty to invest in BPT in view of the social responsibility they need to take. But of equally crucial importance is the role of the competent public authorities to create the appropriate regulatory and legal framework to stimulate companies to invest in BPT. Political representatives and lawmakers have an inevitable, logical responsibility to support health care and welfare. That is what they are for. We identified a shared responsibility making BPT accessible for patients in need. The development and production of BPT products in a pharmaceutical context (clinical trials, production requirements, marketing authorization procedures...) requires time. Patients in need have no time. Therefore, lawmakers and regulators need to design appropriate solutions on a short term to buffer for the years needed for companies to develop BPT-based medicinal products. Much more urgent and optimal, regulatory solutions need to be created to allow hospitals to adopt patient-oriented and tailored BPT in a legal way for treating those patients that are waiting to be cured.

## Figures and Tables

**Table 1:** Six criteria for a therapy to be labelled as an EJMT.

It is morally permissible to set aside the *prima facie duty* not to impose a risk of harm if and only if
(i) there is a just cause;
(ii) those who want to put aside the duty not to impose a risk of harm have good intentions;
(iii) there is a reasonable chance that the just cause will be realized;
(iv) the harm prevented will outweigh the risk of harm imposed;
(v) the just cause cannot be obtained with at least the same probability of success but without imposing a risk of harm;
(vi) those who decide on putting aside the duty not to impose a risk of harm constitute a legitimate authority.
